# Exhaustion or fatigability may not only be cardiac but also myopathic

**DOI:** 10.1007/s12471-015-0682-9

**Published:** 2015-04-14

**Authors:** J. Finsterer, C. Stöllberger

**Affiliations:** KAR, 1030 Vienna, Austria

To the Editor,

With interest, we read the article by Yaksh et al. about a patient with left ventricular hypertrabeculation/noncompaction and Wolff–Parkinson–White syndrome who also manifested with ventricular tachycardia. We have the following comments and concerns.

The patient described is not the first adult with noncompaction who also presented with Wolff–Parkinson–White syndrome. In a previous report, one of the two noncompaction patients from the same family had pre-excitation on electrocardiography [1]. The combination of noncompaction and pre-excitation was also reported in two adult Chinese patients [2]. Additionally, Wolff–Parkinson–White syndrome was described by Moceri et al. and Brembilla-Perrot et al. in a single patient each [3, 4]. In a study of 86 noncompaction patients, Wolff–Parkinson–White syndrome was found in two of them [5].

The authors mention that noncompaction was first described by Engberding et al. [6]. Engberding et al. were indeed the first to recognize and describe this unclassified cardiomyopathy on echocardiography, but there are indications that noncompaction had been described earlier, as indicated in the paper by Feldt et al. in 1969 [7].

Noncompaction is not only associated with electrocardiographic abnormalities such as right bundle branch block, supraventricular tachycardia, or ventricular tachycardia, but any type of electrocardiographic abnormality may be observed in these patients including atrial fibrillation, atrioventricular block, left bundle branch block, or QT prolongation [5].

Easy fatigability or exhaustion may not only be a cardiac symptom but may also be a clinical manifestation of a neuromuscular disorder, frequently associated with noncompaction [8]. Was the patient investigated for neuromuscular disorders by a neurologist? Did she present with any other feature of a neuromuscular disorder, such as muscle weakness, wasting, reduced tendon reflexes, muscle aching, muscle cramping, double vision, or ptosis?

Not only symptomatic patients are investigated for noncompaction but also asymptomatic patients, as reported in a number of cases and family studies. Occasionally noncompaction is found in asymptomatic relatives of symptomatic noncompaction cases. In a family study of 25 relatives of noncompaction patients, asymptomatic noncompaction was detected in four of these relatives [9].

Surprisingly, the authors mention in the discussion that atrial fibrillation could have been the cause of ventricular tachycardia in their patient [6]. Did the patient receive oral anticoagulation in addition to ablation and implantable cardioverter defibrillator implantation as a prophylactic measure? It is essential to put noncompaction patients on oral anticoagulation as soon as atrial fibrillation or heart failure has been documented.

How often did the implantable cardioverter defibrillator discharge during follow-up and which was the arrhythmia that caused the implantable cardioverter defibrillator to react?

In the introduction the authors mention that the clinical presentation of noncompaction includes heart failure, thromboembolic events, or arrhythmias [6]. However, noncompaction patients may present with other cardiac manifestations, particularly if noncompaction is associated with functional or morphological abnormalities in addition to noncompaction (non-isolated noncompaction) [10].

Overall, there is a need to thoroughly investigate and describe noncompaction patients, to screen noncompaction patients for neuromuscular disorders or chromosomal abnormalities, and to screen other family members for noncompaction. For clarification of the pathogenetic background of noncompaction, it is useful to search for any genetic abnormality that may explain the occurrence of noncompaction.

## Funding

None.

## Conflict of interest

None declared.
